# Changes in Soil Microbial Communities Induced by Biodegradable and Polyethylene Mulch Residues Under Three Different Temperatures

**DOI:** 10.1007/s00248-024-02420-0

**Published:** 2024-07-31

**Authors:** Ida Romano, Valeria Ventorino, Mariachiara Schettino, Giuseppina Magaraci, Olimpia Pepe

**Affiliations:** 1 Department of Agricultural Sciences, University of Naples Federico II, Naples, Italy; 2 Task Force On Microbiome Studies, University of Naples Federico II, Naples, Italy

**Keywords:** Mulching, Biodegradable, Soil microbial community, Polyethylene mulches, Mater-Bi mulches

## Abstract

Mulching is a common method increasing crop yield and achieving out-of-season production; nevertheless, their removal poses a significant environmental danger. In this scenario, the use of biodegradable plastic mulches comes up as a solution to increase the sustainability of this practice, as they can be tilled in soil without risk for the environment. In this context, it is important to study the microbial response to this practice, considering their direct involvement in plastic biodegradation. This study evaluated the biodegradation of three commercial mulch residues: one conventional non-biodegradable mulch versus two biodegradable ones (white and black compostable Mater-Bi mulches). The experiment was conducted under three incubation temperatures (room temperature 20–25 °C, 30 °C, and 45 °C) for a 6-month trial using fallow agricultural soil. Soil without plastic mulch residues was used as a control. White mater-bi biodegradable mulch residues showed higher degradation rates up to 88.90% at 30 °C, and up to 69.15% at room temperature. Furthermore, incubation at 45 °C determines the absence of degradation for all types of mulch considered. Moreover, bacterial alpha diversity was primarily influenced by plastic type and temperature, while fungal populations were mainly affected by temperature. Beta diversity was impacted by all experimental variables. Predicted functional genes crucial for degrading complex substrates, including those encoding hydrolases, cutinases, cellobiosidases, and lipases, were derived from 16S rRNA gene sequencing data. Cluster analysis based on predicted enzyme-encoding gene abundance revealed two clusters, mainly linked to sampling time. Finally, core microbiome analysis identified dominant bacterial and fungal taxa in various soil-plastic ecosystems during degradation, pinpointing species potentially involved in plastic breakdown. The present study allows an assessment of how different temperatures affect the degradation of mulch residues in soil, providing important insights for different climatic growing zones. It also fills a gap in the literature by directly comparing the effects of biodegradable and polyethylene mulches on soil microbial communities.

**Supplementary Information:**

The online version contains supplementary material available at 10.1007/s00248-024-02420-0.

## Introduction

Depending on the durability, flexibility, and cost-effectiveness of plastics and their wide range of uses, 391 million tonnes (Mt) of it was produced globally only in 2021 [1]. The increase in plastic manufacture and use has two primary drawbacks. (i) Plastics are expected to account for 15% of the world’s carbon emissions by 2050; (ii) post-consumer plastics are a significant source of environmental plastic pollution and marine litter [2]. Plastic most probably accessed the soil environments due to increase of petroleum-based consumer products like synthetic fibers [3]. Nowadays, the ways by which plastics enter agricultural, horticultural, orchard, grassland, and forest soils comprise different pathways: the spreading of sewage sludge, composted and fermented organic waste, the plowing of mulching film, and irrigation with contaminated water [3, 4]. In addition to these pathways, agriculture’s reliance on the plastic market is growing: the estimated rate of plastic use in agriculture is expected to rise by about 64% by 2030 to meet the growing population demand for food [1]. Soil plastic pollution can alter its structure, adversely affect microbial communities, and transport chemical contaminants [2, 5–7]. In addition, microplastics can be uptake by plants and enter the food chain, raising concerns about food safety [2, 8]. Mulching has been identified as one of the pathways for plastic input in soil. It is a common practice for cultivation of horticultural crops that significantly boost their production and quality [9]. These films are widely used as they help to maintain soil moisture, control weed growth, and to modulate effect on soil temperature, satisfying the need of more sustainable agriculture systems, as it is considered a practice for saving soil and water [9, 10].

Polyethylene (PE) or low-density polyethylene (LDPE) mulch plastic films are typically used in agriculture, for example, for soil solarization. Nevertheless, since they require over 100 years to decompose, they have a significant negative influence on the environment [11]. Their disposal methods include burning, burying, and recycling [12, 13]. However, because of soil contamination, recycling can be challenging and costly [14]. The use of biodegradable plastic mulches (BDMs) is an attractive eco-sustainable alternative approach to overcome the environmental pollution problems caused by the use of plastic films [15], because they can be degraded progressively in the soil without releasing toxic residues [16]. After the growing season, PE or LDPE films must be removed from the soil surface. In contrast, BDMs can be tilled into the soil where they are biodegraded by micro-organisms. BDM fragments contribute physically and biogeochemically to the soil. This is characteristic to BDMs, so research on non-biodegradable polymers cannot provide insights into the effects of BDMs on soil ecology and function [17].

BDMs, tilled into soil, provide a carbon input; several studies showed that soil microorganisms respond to these inputs even if tiny, especially in agricultural soil where microbial development is carbon limited. Previous research demonstrated that BDMs increased microbial biomass, and enzyme activity [6, 18], and altered soil microbial community structure [5, 7]. The addition of residual plastic film in soil seemed to harm the bacterial community’s richness and diversity on plastic surfaces, but it did not influence on the surrounding soil community [19]. Other studies found increased fungus abundances in soil as a result of BDM incorporation [6, 7]. Nevertheless, the microbial response to BDMs is influenced by environment, soil type, and/or management practices. Confirming this, Li et al. [6] find enrichment of fungus in one location and of Gram-positive bacteria in another.

On the other hand, the action of microorganisms on biodegradable polymers in soil is well known and can be simplified into three steps: (i) microbial colonization of the polymer surface, (ii) enzymatic depolymerization of polymers, and (iii) microbial assimilation and utilization of the monomers and oligomers released from polymers by enzymatic hydrolysis [20]. Other enzymes typically related to plastic breakdown include those from the esterase family, both bacterial and fungal, such as cutinases and lipases [20]. Temperature is a key factor in this process. Effective biodegradation of plastics in soil by microorganisms occurs within the mesophilic temperature range (10 to 45 °C), which promotes optimal microbial activity [21]. Additionally, the EN 17033 standard for biodegradable mulch films suggests a temperature around 25 °C to effectively represent conditions favorable for mesophilic soil microorganisms [21, 22]. In the context of climate change, rising temperatures—projected to increase by up to 2 °C by the end of the century—along with changes in rainfall patterns leading to more frequent and severe droughts, can indirectly impact microbial communities by increasing evapotranspiration rates, reducing soil moisture, and thereby affecting their functions [23] and consequently plastic biodegradation.

In this scenario, microbial populations emerge as pivotal players in the degradation of plastic fragments within the soil environment. Critical gaps remain in our understanding effects of BDMs on soil ecosystems; also due to the absence of studies including a direct comparison of PE with BDMs to determine whether they affect soils differently.

The presented study aimed to assess the degradation potential and microbial dynamics associated with two biodegradable Mater-Bi mulches and traditional low-density polyethylene mulch residues when mixed in fallow agricultural soil under three temperature conditions. Evaluation of plastic degradation involves separating and weighing large plastic fragments at the end of the experiment. Furthermore, high-throughput sequencing was used to analyze changes in bacterial and fungal communities throughout, and the predicted functions of the trials, shedding light on the intricate interactions between plastic mulches and soil microbiota.

## Materials and Methods

### Soil Samples and Plastic Mulch Residual Preparation

Fallow agricultural soil samples (0–5-cm depth; pH-H_2_O 7.21; electrical conductivity 263.5 μS cm^−1^; CaCO_3_ 4%; organic matter 9.30%; organic carbon 5.39%; total nitrogen 0.47%; C/N ratio 11.42%; phosphorus available 475.16 ppm; potassium exchangeable 716.87 ppm; Supplementary Material) were collected during spring season (March 2022) in Castellammare di Stabia (Naples; 40°41′15″N; 14°29′35″E), an area under a Mediterranean climate (average annual precipitation of approx. 52.20 mm, temperature from 7 to 32 °C). A total of 4.5 kg of soil underwent drying at room temperature and sieving through a 2-mm mesh to segregate sand, silt, and clay from larger particles, according to Al Hosni et al. [24].

### Assembly of Soil-Plastic Ecosystem

The study employed three mulch sheets including two BDMs, white compostable Mater-Bi (MB, 15 µm), black compostable Mater-Bi (TMB, 20 µm) mulches, and a traditional black low-density polyethylene mulch (LDPE, 30 µm). The two biodegradable film Mater-Bi® by Novamont Company are starch-based, treated with biodegradable polyesters, and certified compostable (OK Biodegradable Soil by TÜV) [25], according to European standards (UNI EN 13432:2002, UNI EN 14995). In contrast, traditional mulching plastic film is made from low-density polyethylene resin pellets, offering easy processability, chemical resistance, durability, and flexibility [26]. All sheets were cut into small fragments less than or equal to 2 cm^2^ according to the procedure described by Al Hosni et al. [24].

A mixture of plastic and soil containing 1% w/w plastic per 100 g of soil was placed in transparent magenta boxes (76 × 76 × 102 mm, Magenta GA-7–3 Plant Culture Box). Magentas containing soil and plastic were incubated at three different temperatures: room temperature (RT; 20–25 °C), 30 °C, and 45 °C, selected on the basis of the guidelines of standard test methods for biodegradable mulch films [22]. The experiment was conducted with five replicates per treatment. Soil samples without plastic were used as controls.

The magenta boxes were placed in humid chambers (RH 70%) for 6 months. They were weighed every 2 days to assess water evaporation, and the lack water was compensated spraying tap water.

Sampling of the soil-plastic mixture was performed at the beginning (t0), after 3 (t3), and 6 (t6) months of incubation. The collected samples were stored at − 20 °C until molecular analysis.

### Quantification of Degraded Plastics

At the end of the 6-month trial, plastics residuals were quantified for each soil-plastic mixture. After final sampling, each mixture was dried at 50 °C for 72 h and sieved through a 2-mm mesh sieve to collect plastic fragments larger than 2 mm. This fraction was first rinsed with sterile distilled water to remove adhering soil particles, after drying it was weighed to calculate the residual percentage of plastic, using the following equation [27]:

$$D = ((Mi-Mf))/{Mi }^{*} 100$$

where *D* is the percentage of plastics degraded at the end of the test, *Mi* is the starting dry mass of plastic fragments, and *Mf* is the dry mass of recovered plastic fragments > 2 mm.

Plastic particles smaller than 2 mm represent the proportion of degraded plastic.

### DNA Extraction, Amplicon Sequencing, and Data Processing

For molecular analysis, total genomic DNA was extracted from soil-plastic ecosystem using a FastDNA SPIN Kit for Soil (MP Biomedicals, Illkirch Cedex, France) according to the manufacturer’s instructions.

#### High-Throughput Sequencing

Synthetic oligonucleotide primers S-D-Bact-0341F50 (5′-CCTACGGGNGGCWGCAG-3′) and S-D-Bact-0785R50 (5′-GACTACHVGGGTATCTAATCC-3′) [28], and the primers EMP.ITS1 (5′-CTTGGTCATTTAGAGGAAGTAA-3′) and EMP.ITS2 (5′-GCTGCGTTCTTCATCGATGC-3′) [29] were used to evaluate bacterial and fungal diversity, respectively, by amplicon-based metagenomic sequencing. PCR conditions for V3–V4 region consisted of 25 cycles (95 °C for 30 s, 55 °C for 30 s, and 72 °C for 30 s) plus one additional cycle at 72 °C for 10 min as a final chain elongation. PCR conditions for ITS1–ITS2 region consisted of 35 cycles (94 °C for 30 s, 52 °C for 30 s, and 68 °C for 30 s) plus one additional cycle at 68 °C for 7 min as a final chain elongation. Agencourt AMPure beads (Beckman Coulter, Milan, IT) were used to purify PCR products, whereas quantification was performed by AF2200 Plate Reader (Eppendorf, Milan, IT). Equimolar pools were obtained, and sequencing was carried out on an Illumina MiSeq platform, yielding to 2 × 250 bp, paired end reads. The raw Illumina sequencing data are available in the Sequence Read Archive Database of the National Center of Biotechnology Information (PRJNA1127654).

#### Bioinformatics and Data Analysis

After sequencing, QIIME 2 software was used to analyze fastq files [30]. Sequence adapters and primers were trimmed by using a cut adapter, whereas DADA2 algorithm [31] was used to trim low-quality reads, remove chimeric sequences, and joined sequences shorter than 250. DADA2 produced amplicon sequence variants (ASVs), which were rarefied at the lowest number of sequences/sample and used for taxonomic assignment using the QIIME feature-classifier plugin against Greengenes and UNITE database for the bacterial and fungal microbiota, respectively. The taxa abundances were recalculated after the exclusion of chloroplast, mitochondria contaminants, and singleton ASVs.

### Statistical Analysis

Data on degraded plastic amounts were analyzed by univariate ANOVA, followed by Tukey’s HSD post hoc for comparison of means (*p* < 0.05) using IBM SPSS 19.0 statistical software package (SPSS Inc., Cary, NC, USA).

The R statistical environment (R version 4.1.2) was used for sequencing data analysis and data visualization using RStudio [32]. Microbial community data were organized and analyzed with R package phyloseq [33] and vegan 2.5–6 [34]. The quality of sequencing was controlled with rarefaction analysis using the rarecurve function (vegan package). The Shannon–Weaver index (*H*), used to assess alpha diversity, was calculated as follows: *H* = -sum *pi* * ln *pi*, where *pi* is the proportional abundance of species *i*. The diversity was calculated as follows: *D* = exp(*H*) [35]. Beta diversity was examined by permutational multivariate analysis of variance (PERMANOVA) using the adonis function from vegan. Principal coordinate analysis (PCoA) on Bray–Curtis dissimilarities was used to visualize the differences between samples. To visualize microbial community structure, unconstrained ordination by principal component analysis (PCA) on clr-transformed ASVs tables was used, followed by distance-based redundancy analysis (db-RDA), constrained for statistically significant factors identified in PERMANOVA and conditioning for block. The metabolic function was predicted by Tax4Fun analysis through the Kyoto Encyclopedia of Genes and Genomes (KEGG) database [36, 37]. The analysis focused on the differences in predicted abundances of enzyme‐encoding genes linked with plastic degradation. ANOVA (*p* ≤ 0.05) was used to assess the difference in predicted abundance considering the experimental factors. Heatmaps were generated in R using the package pheatmap 1.0.12 [38]. Venn diagrams were created to assess the unique and shared core microbiota, identifying overlap between soils treated with different plastic mulch residues (MB, TMB, LDPE) at RT and 30 °C (detection > 0.01% and prevalence = 70% for bacteria; detection > 0.01% and prevalence = 99% for fungi, based on prevalence plot). Finally, ASV sequences of the core were compared with the GenBank nucleotide data library using BLAST software on the National Centre for Biotechnology Information website (ASVs belonging to the same species were collapsed).

## Results

### Effect of Temperature on Plastic Degradation

**Fig. 1:**
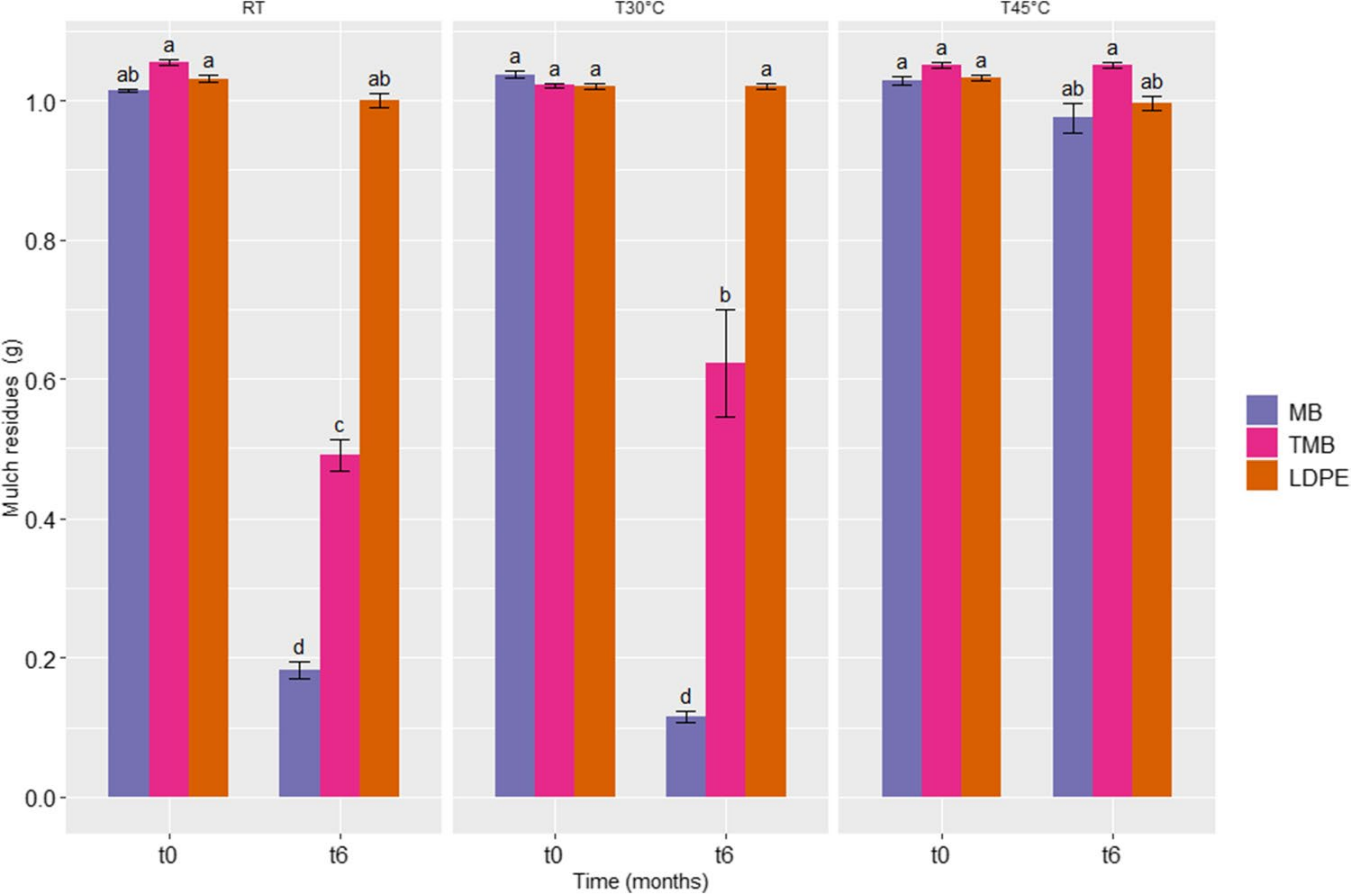
Comparison of mulch residues (g) of biodegradable Mater-Bi (MB and TMB) and non-biodegradable (LDPE) plastic mulch films at the beginning (t0) and after 6 months of degradation in soil (t6), at the three incubation temperatures (RT, T30°C, T45°C). Different letters indicate statistically significant differences (univariate ANOVA and Tukey’s HSD post hoc, *p* < 0.05). The error bars represent the means ± SE of five replicates

After 6 months of incubation of soil, plastic mulch residues greater than 2 mm were weighed for comparison with the initial weight (Fig. 1, Supplementary Table 1). The incubation temperature used in the experiment influenced the degree of degradation of the plastic mulching films. At RT, biodegradable mulch residues, MB and TMB, showed a high percentage of degradation (69.15% and 51.36% respectively). The increase of temperature at 30 °C, allowed an increase of the MB mulch film residues degradation (88.90%), and a decrease of TMB degradation to 38.86%, while at 45 °C no biodegradation activity, was observed. After 6 months, no degradation of LDPE was observed at all temperature assayed (Fig. 1, Supplementary Table 1).

### Dynamic of Microbial Communities in Soil with Mulching Films at Different Temperatures

#### Dynamic of Bacterial Communities

**Fig. 2:**
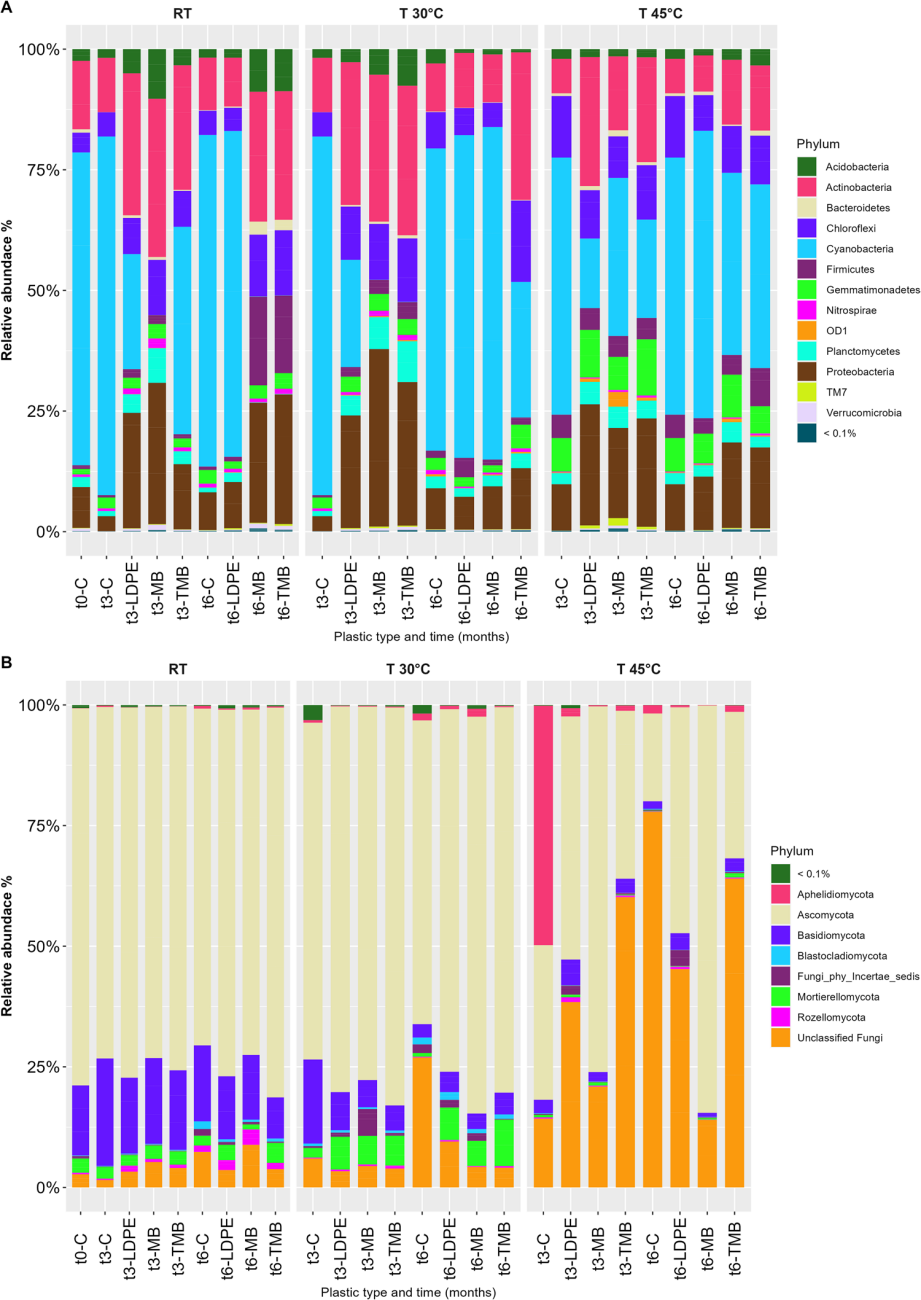
Relative abundance of bacterial (**A**) and fungal (**B**) phyla for soil samples containing biodegradable Mater-Bi (MB and TMB) and non-biodegradable (LDPE) plastic mulch films at the beginning (t0), after 3 (t3), and 6 (t6) months of incubation at different temperatures (room temperature RT, 30 °C, 45 °C). Control soil samples without plastic (**C**) were analyzed in parallel. Only ASVs at phyla level with an incidence > 0.1% are shown in the legend

Amplicon sequencing yielded a total of 38,723 bacterial ASVs obtained from 61 samples. Cyanobacteria, Acidobacteria, Actinobacteria, Bacteroidetes, Chloroflexi, Firmicutes, and Proteobacteria were the dominant groups at the beginning of the process (Fig. 2A). Cyanobacteria decreased significantly over time and with different mulches (*p* < 0.01 and *p* < 0.05, respectively; Supplementary Table 4). After 3 months at RT, Cyanobacteria decreased in MB-soil (~ 68.84 to 0.01%) and in LDPE-treated soil (64.84 to 23.85%). At 30 °C, their abundance decreased up to 22.23% in LDPE-treated soil. Acidobacteria were significantly affected by temperature and mulch type (*p* < 0.001 and *p* < 0.05, respectively; Supplementary Table 4). In MB-soil at RT, Acidobacteria increased from 2.42 to 10.28% after 3 months, while they decreased up to 0.77% in LDPE-treated soil. At 30 °C, their abundances were 5.29% in MB and 7.57% in TMB soils after 3 months of incubation. Actinobacteria abundances were significantly influenced by all variables (Supplementary Table 4). They increased in MB-soil at RT from 14.20 to 32.81% and in LDPE-treated soil up to 29.43%. At 30 °C, Actinobacteria levels were around 30.00% in MB, TMB, and LDPE soils. Chloroflexi were one of the dominant groups in the experimental process, but their abundance was not significantly influenced by any of the experimental variables (Supplementary Table 4). Firmicutes were significantly affected by time and temperature (*p* < 0.01; Supplementary Table 4). Their abundance in MB-soil at RT increased from ~ 1 to 18.39%, and slightly in LDPE-treated soil. At 30 °C, Firmicutes levels were around 2–3% in MB, TMB, and LDPE soils. Proteobacteria, influenced by both time and plastic type (*p* < 0.01 and *p* < 0.05, respectively; Supplementary Table 4), increased in MB-soil at RT from 8.47 to ~ 29.29% and in LDPE-treated soil to 23.93%. At 30 °C, their levels were around 30–37% in MB, TMB, and LDPE soils. Planctomycetes, affected by time and temperature (Supplementary Table 4), decreased in MB-soil at RT from 7.15 to 0.08%, while were quite stable in LDPE-treated soil. At 30 °C, their levels were 6–8% in MB, TMB, and LDPE soils. Gemmatimonadetes were significantly affected by temperature (*p* < 0.001; Supplementary Table 4). At 30 °C, they were about 3.5% in MB and TMB soils, and increased in LDPE-treated soil from 1.14 to 3.19%. At 6 months, LDPE-treated soil at RT had a stable Gemmatimonadetes population of around 3%. At 45 °C, the bacterial composition of all analyzed soil samples remained quite stable for up to 6 months. Slight changes were observed after 3 months of incubation due to a decrease of Cyanobacteria and an increase of Actinobacteria, Proteobacteria, Chloroflexi, Gemmatimonadetes, Firmicutes, and Planctomycetes. After 6 months, the bacterial composition was similar to the control after 3 months of incubation (Fig. 2A).

#### Dynamic of Fungal Communities

Amplicon sequencing yielded 11,537 fungal ITS ASVs. At RT and 30 °C, the phylum Ascomycota was dominant with relative abundances ranging from 62.96 to 82.41%, followed by the phylum Basidiomycota (from ~ 3.21 to 22.26%). In addition, the phylum Mortierellomycota (from 1.03 to 9.64%) and unclassified fungal populations (from 4 to 27%) were present in all samples (Fig. 2B). These four phyla were also significantly influenced by the addition of plastic to the soil (*p* < 0.05; Supplementary Table 4).

At 45 °C, there was a shift in fungal community with an increase of unclassified fungi (from 13.99 to 77.86%), followed by a decrease in the phyla Basidiomycota (ranging from 0.83 to 5.31%) and Ascomycota (ranging from 18.20 to 84.42%).

### Microbial Diversity

**Fig. 3:**
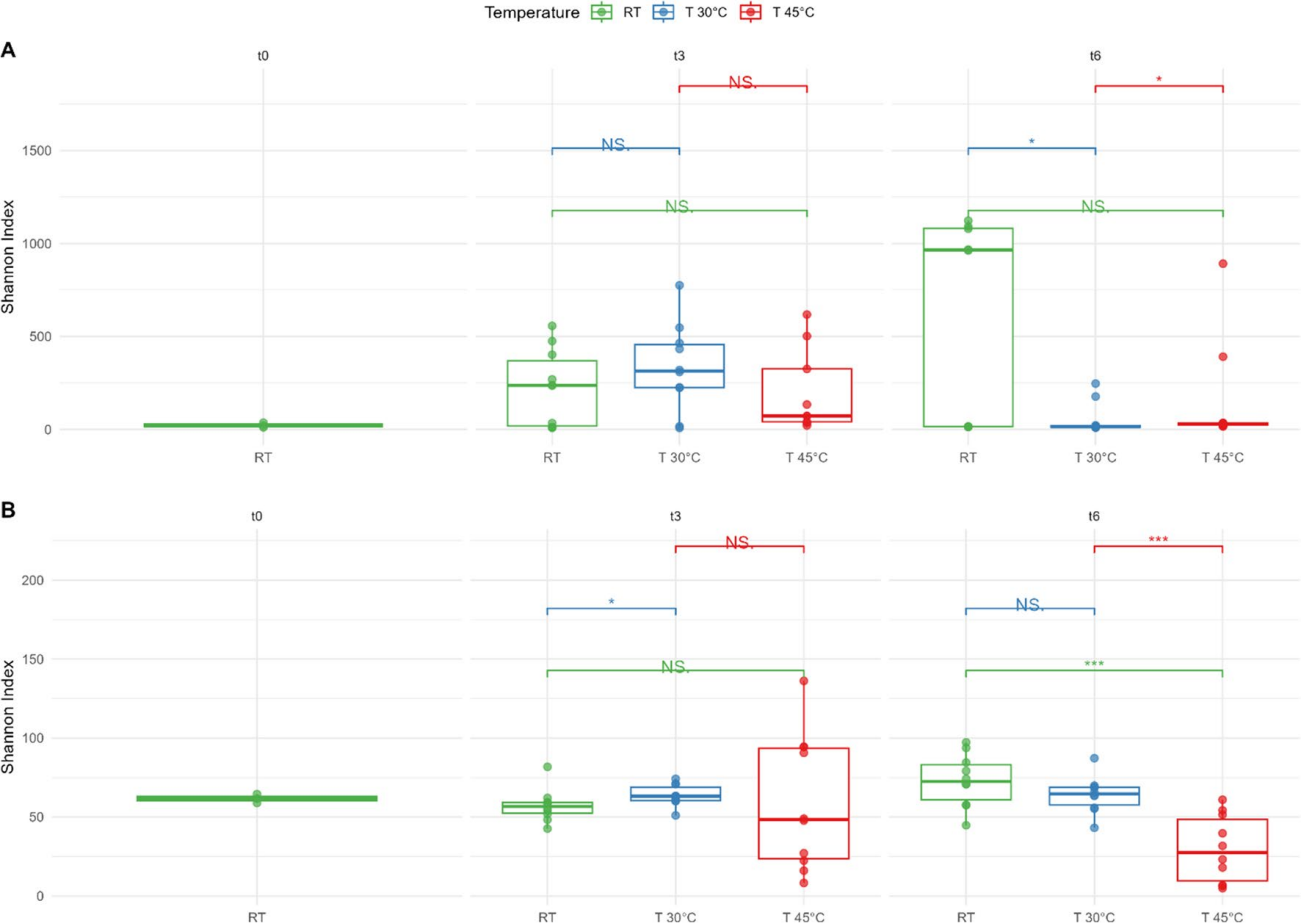
Alpha diversity of (**A**) bacterial and fungal (**B**) communities in soil samples containing biodegradable Mater-Bi (MB and TMB) and non-biodegradable (LDPE) plastic mulch films at the beginning (t0), after 3 (t3), and 6 (t6) months of incubation at different temperatures RT (green), 30 °C (blue), and 45 °C (red). Control soil samples without plastic (**C**) were analyzed in parallel. Asterisks denote statistical significant differences given by *T*-test (0 “***” 0.001 “**” 0.01 “*” 0.05 “.” 0.1 “” 1)

Analysis of variance of Shannon’s index for mulch plastic-type, sampling time, and temperature showed that bacterial diversity was affected by mulch type and temperature, whereas fungal diversity was affected only by temperature (Supplementary Tables 2A and 2B). After 6 months, significant differences were found between soil incubated at RT and at 30 °C, and also between 30 and 45 °C (Fig. 3A). The Shannon index of the fungal community showed significant differences between soils incubated at RT compared to 30 °C after 3 months (Fig. 3B), whereas after 6 months, both soils incubated at RT and 30 °C differed from 45 °C (*p* < 0.05; Fig. 3B). Furthermore, a higher Shannon index was found in MB and TMB-treated soil compared with soil added with LDPE mulch residues as well as control (*p* < 0.05; Supplementary Fig. 1).

**Fig. 4:**
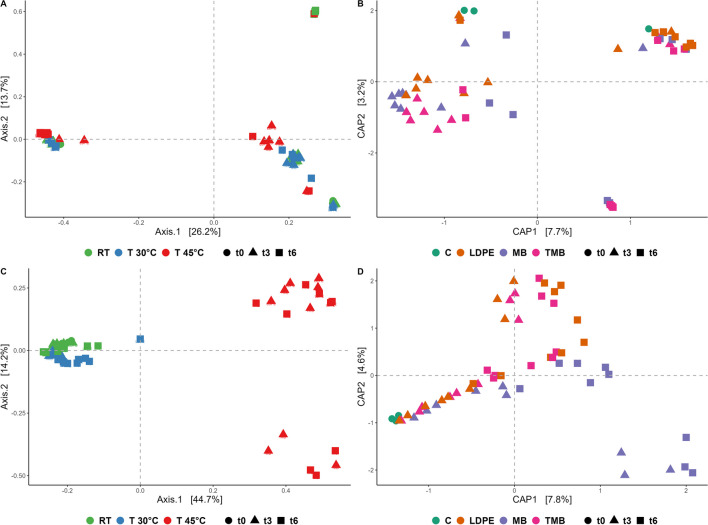
Beta diversity assessed with Bray–Curtis distance of bacterial (**A**) and fungal (**C**) community at RT (green), 30 °C (blue), and 45 °C (red), at different sampling times t0 (circle), t3 (triangle), and t6 (square). Constrained distance-based redundancy analysis (db-RDA) of bacterial (**B**) and fungal (**D**) community composition with different plastic soil assembled systems, control (dark green), LDPE (orange), MB (violet), and TMB (pink) at different sampling times based on significant factors identified in PERMANOVA

PERMANOVA analysis on beta diversity performed on all the experimental factors allows to state that each variable affects the bacterial and fungal community structures (Fig. 4A and 4C; Supplementary Table 3). Based on the statistical analysis, a constrained ordination (db-RDA) was performed for both plastic type and sampling time (*p* < 0.05) for bacterial and fungal communities (Fig. 4B and 4D). No distinct separation pattern could be observed for bacterial community (Fig. 4A and 4B). Fungal communities showed temperature-specific clustering (Fig. 4C), constrained ordination revealed a distinct trend in soil amended with MB mulch residues (Fig. 4D).

### Functional Prediction Analysis

Functional profiles were predicted from the 16S rRNA gene sequencing data to compare soil treated with different mulch plastic residuals. While over 6000 functional genes were predicted, the focus was on enzyme-encoding genes like hydrolases, lipases, cutinase, cellobiosidase, and catalases. ANOVA analyses pinpointed 57 predicted genes significantly affected by the experimental factors (*p* < 0.05; Supplementary Table 5).

**Fig. 5:**
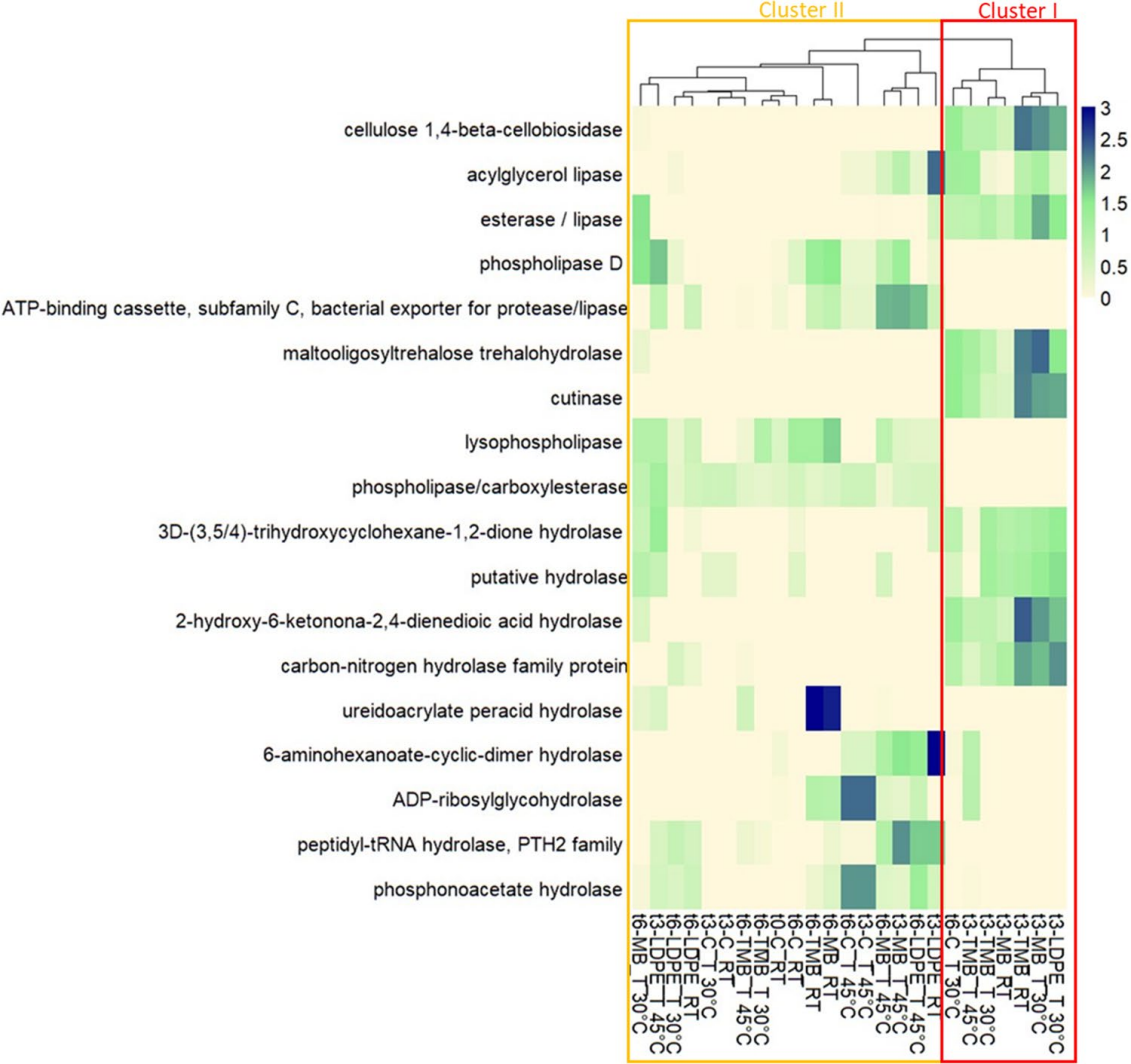
Predicted abundances of selected cutinase, cellulase, hydrolase, and lipase enzyme‐encoding genes predicted through Tax4fun R package. The color code refers to gene abundance, with high predicted abundances (blue) and low predicted abundances (light yellow) in soil samples containing biodegradable Mater-Bi (MB and TMB) and non-biodegradable (LDPE) mulch films at the beginning (t0), after 3 (t3), and 6 (t6) months of incubation at different temperatures (room temperature RT, 30 °C, 45 °C). Control soil samples without plastic (C) were analyzed in parallel

After identifying the encoding genes predicted abundances significantly affected by experimental variables, the pool of encoding genes was further narrowed down. Only genes associated with the degradation of complex substrates, including cellulose, starch, and other organic compounds, were investigated (Fig. 5) along with catalase as biological indicator of soil health and productivity (Supplementary Fig. 2). Identified enzymes linked with plastic degradation included cellulase, lipases, esterases, and hydrolases. Two main clusters resulted from the functional profiles of the bacterial communities, which were mostly associated with the sampling time (Fig. 5). The first cluster (cluster I) includes soil samples with MB and TMB mulch plastic residues collected after 3 months of incubation at 30 °C and RT. This cluster also contains soil samples enriched in TMB and LDPE residues after 3 months at 45 °C and 30 °C, along with one control sample (C) after 6 months at 30 °C (Fig. 5). The most abundant enzyme‐encoding genes in these samples were 1,4-beta-cellobiosidase (K01225), acylglycerol lipase (K01054), esterase/lipase (K01066), maltooligosyltrehalose trehalohydrolase (K01236), cutinase (K08095), 3D-3,5/4-trihydroxycyclohexane-1,2-dione hydrolase (K03336), putative hydrolase (K04477), 2-hydroxy-6-ketonona-2,4-dienedioic acid hydrolase (K05714), and carbon–nitrogen hydrolase family protein (K08590).

The second cluster (cluster II) includes MB samples incubated at 45 °C, soil supplemented with LDPE residues incubated at RT and 45 °C, and samples incubated at 45 °C after 6 months. The most abundant enzyme-encoding genes in cluster II were phospholipase D (K01115), lysophospholipase (K01048), phospholipase/carboxylesterase (K06999), 6-aminohexanoate cyclic dimer hydrolase (K01471), ADP-ribosylglycohydrolase (K05521), peptidyl-tRNA hydrolase (pth2 family) (K04794), and phosphonoacetate hydrolase (K06193). Lastly, ureidoacrylate peracid hydrolase increased after 6 months in two soil samples supplemented with MB and TMB and incubated at RT. The predicted catalase gene EC.1.11.1.6. was affected by the addition of plastic in soil (*p* < 0.05), whereas catalase-peroxidase EC.1.11.1.21. by time (*p* < 0.01; Supplementary Table 4). The predicted abundance of the catalase gene increased after 3 months in the plastic-enriched samples at both RT and 30 °C (cluster II; Supplementary Fig. 2). Notably, catalase-peroxidase also increased after 3 months in all the biodegradable mulch-enriched samples (Supplementary Fig. 2).

### Core Microbiota

The microbial core was calculated in soil samples associated with the degradation of mulch residuals at RT and 30 °C (Table 1 and Supplementary Fig. 3). The bacterial core consists of 84 ASVs for bacteria and 45 for fungi. Among the three plastic types, four ASVs were shared among bacteria and 30 among fungi (Supplementary Fig. 2). ASVs collapse within their respective taxa led to a reduction in taxonomic diversity. *Thermoanaerobaculum aquaticum* MP-01, *Arthrobacter nitrophenolicus* SJCon, *Pseudarthrobacter phenanthrenivorans* Sphe3, *Sphaerobacter thermophilus* DSM 20745, and *Neobacillus endophyticus* BRMEA1 were detected only in the microbiota of soil with MB degradation (Table 1, Supplementary Fig. 2A). Other species present only in soils with LDPE belonged to the phyla Actinobacteria, Firmicutes, Gemmatimonadetes, and Proteobacteria (Table 1). Soil with TMB was characterized by three species *Dehalogenimonas alkenigignens* IP3-3, *Thermoflavimicrobium daqui* FBKL4.011, and *Hydrogenispora ethanolica* LX-B, shared with soils containing MB and LDPE (Table 1, Supplementary Fig. 3A).

**Table 1:**
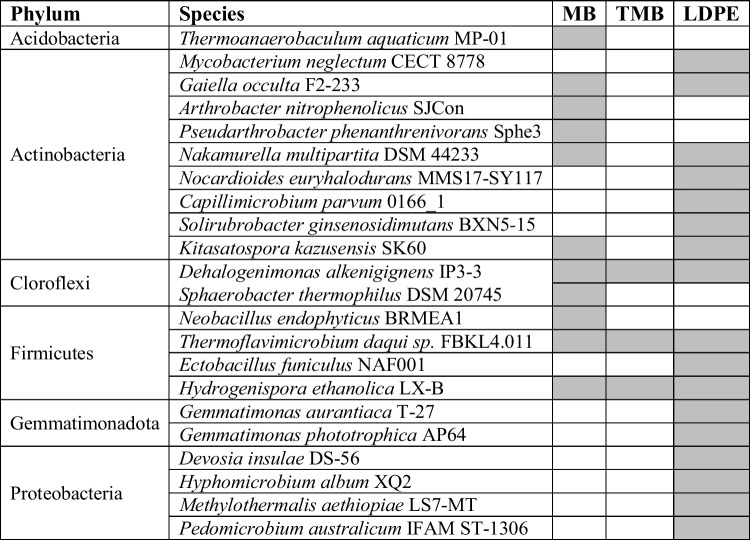
Bacterial species representing the core (70% of prevalence) of different soil samples containing biodegradable Mater-Bi (MB and TMB) and non-biodegradable (LDPE) ) plastic mulch films at RT and 30°. Species obtained by comparing ASV sequences with the GenBank nucleotide data library via *Blastn*. ASV sequences belonging to the same species were collapsed.)

All fungal shared core members of the TMB-treated soil were in common with the other two mulch types (Table 2, Supplementary Fig. 3B). Between them, *Lophiotrema rubi*, unclassified *Nectriaceae*, and unclassified *Agaricaceae* were shared with both MB and LDPE soils. *Cheilymenia* sp. and *Solicoccozyma aeria* were common between TMB and MB-treated soils, whereas TMB and LDPE shared *Gibberella* sp*.*, *Preussia flanaganii*, and *Preussia flanaganii*. Finally, *Metacordyceps chlamydospore*, *Niesslia mucida*, unclassified *Fusarium*, and unclassified *Mortierellaceae* were detected only in the core microbiota of LDPE-treated soils (Table 2).

**Table 2:**
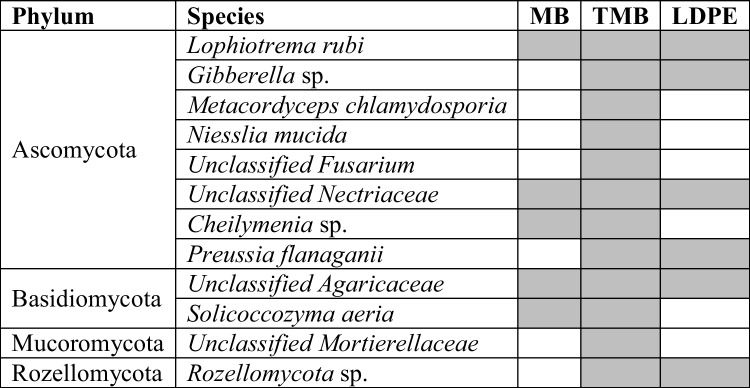
Fungal species representing the core (99% of prevalence) of different soil samples containing biodegradable Mater-Bi (MB and TMB) and non-biodegradable (LDPE) mulch films at different incubation temperatures: room temperature and 30 °C. Species obtained by comparing ASV sequences with the GenBank nucleotide data library via *Blastn*. ASV sequences belonging to the same species were collapsed

## Discussion

### Plastic Degradation

The findings of this study provide important insights into the biodegradation dynamics of various mulch films across different temperatures. The significant degradation of biodegradable plastic mulch films (MB and TMB) during 6 months at RT demonstrates their promise as long-term alternatives to non-biodegradable options (LDPE). The biodegradation of TMB by 51.36% and MB mulch by 69.15% indicates their ability to decompose over time, concerning LDPE mulch recalcitrant to biological decomposition during the 6-month trial, according to previous establishment [11]. The impact of temperature on degradation rates is a pivotal consideration. The rapid breakdown seen at 30 °C, particularly with MB mulch (88.90%) as well as no degradation at 45 °C, indicates that temperature impact on biodegradation efficiency is linked to microbial catabolic activity. Sintim et al. [39] observed temperature-dependent variations in mulch film biodegradability under field conditions, with higher temperatures significantly enhancing polymer breakdown. Nevertheless, our study demonstrated that elevating temperatures up to 45 °C markedly affects metabolic activity of mesophilic bacterial populations, corresponding to the non-degradation of all mulch residues [40]. This result marked the temperature’s pivotal role in shaping microbial communities and impacting their capacity to degrade mulch residues [23]. In the context of climate change, rising temperatures could potentially diminish the efficacy of biodegradable mulches in promoting sustainable agriculture. This evidence concerning the impact of temperatures emphasizes the need to consider this environmental variable to optimize the degradation rates based on local climate. Nevertheless, it must be considered that in a real *scenario*, the temperature along with other biotic (variety of microorganisms such as bacteria, fungi, and Archaea) and abiotic factor (e.g., light, oxygen concentrations, humidity, and acidity) affects polymer biodegradability [41, 42].

### Microbial Community Composition and Diversity

This study provides insights into the biodegradation dynamics of various mulch films at different temperatures, comparing the effects of biodegradable (MB and TMB) and polyethylene (LDPE) mulch residues on soil microbial communities. Soil samples treated with MB and TMB showed consistent trends, including reductions in Cyanobacteria and Planctomycetes after 3 months, while Actinobacteria, Proteobacteria, Chloroflexi, Firmicutes, Bacteroidetes, and Acidobacteria dominated the bacterial composition, aligning with existing research on plastics in diverse environments [43–45]. Previous studies highlighted how biodegradable plastics can enrich Proteobacteria, which play crucial roles in soil biogeochemical cycles due to their association with total nitrogen and organic carbon levels [7, 17, 46]. Bacteroidetes, widely distributed in ecosystems, contribute significantly to the degradation of complex organic materials, aided by genera like Flavobacterium that break down polysaccharides and influence denitrification processes [47–49].

Evidence suggests that biodegradable plastics in agricultural soils contribute minimally to carbon levels, affecting soil bacterial responses due to limited carbon availability [17]. Furthermore, main variations were at RT and 30 °C supporting the results of plastic breakdown and highlighting that burial of biodegradable plastics can lead to changes in soil microbial community structures [5–7].

LDPE-treated soil displayed minor changes in bacterial composition, resembling control groups after 6 months, and presenting enriched microplastic-degrading taxa such as Bacteroidetes and Proteobacteria [50, 51].

All samples incubated at 45 °C showed a stable bacterial composition up to 6 months, probably due to bacterial decrease in activity with higher temperature occurrences [40], aligning with the absence of plastic degradation.

No fluctuations in the abundance of predominant fungal phyla Ascomycota, Basidiomycota, and Mortierellomycota were observed at RT and 30 °C. Previous research showed that the phyla Ascomycota and Basidiomycota were responsible for the breakdown of oil-based polymeric polymers [7, 17, 52]. Therefore, these findings suggest that, at least throughout the 6-month trial, no mesophilic fungal communities linked to plastic degradation emerged. Otherwise, the shifts observed at 45 °C in fungal community composition highlight the influence of both plastic and temperature on fungal dynamics, in absence of plastic degradation. In all samples, the Ascomycota and Aphelidiomycota decreased, and the unclassified fungi increased. These last can be assumed that the thermophilic phyla selected by the plastic and temperature are responsible for the shift in fungal profiles at 45 °C. Thermophilic fungi are characterized by their ability to thrive at temperatures ranging from 20 to 62 °C [53]; however, there are an estimated 3 million thermophilic fungal species on Earth, of which only about 100,000 have been identified, so the increase in unclassified ASVs at 45 °C may be due to the presence of species yet to be classified [53].

Analyses of alpha diversity across the entire data set confirmed that the mulch plastic type was the most discriminating factor shaping bacterial communities, followed by temperature that impact also fungal communities. It was interesting to note that the bacterial community responded differentially to each source of plastics tilled in the soil supporting results of plastic degradation. The bacterial Shannon diversity index was higher in soils treated with TMB and MB mulch residues and decreased in LDPE-amended soils as well as in controls. The introduction of available carbon sources for bacteria through biodegradable mulch residues affected the Shannon index, confirming the response of soil microbes to these inputs [17]. According to previous studies [40], the effect of the different temperatures on the microbial community was particularly evident at the end of the experiment, e.g., by reducing the activity of mesophilic bacteria or selecting thermophilic fungi (45 °C).

In addition, the beta-diversity analysis of the microbiota associated with mulch residues, investigated with PERMANOVA, showed the significant influence of sampling times that reflect the temporal dynamics of plastic degradation process. The distinct sampling stages could help to determine the interaction between microorganisms and plastic tilled in the soil. Over time, enzymes break down the plastic and microbes use the resulting fragments for their nutrition by a process involving the turnover of different populations with distinct enzyme kits [20]. Moreover, using biodegradable mulch can benefit the soil’s biogeochemical cycles by promoting a dynamic microbial community, due to increased carbon intake.

### Predicted Enzymatic Activity

In this study, particular focus is placed on genes encoding hydrolases, lipases, cutinase, and cellobiosidase, due to their significant role in natural or synthetic polymer biodegradation [54, 55].

The clustering of predicted functions demonstrated that potential gene abundances were strongly affected by sampling time since two major clusters were observed: (I) samples collected after 3 months of incubation at 30 °C and RT tilled with biodegradable mulch residues (MB and TMB), comprising three exceptions: t3-TMB_T 45 °C, t6-C_T 30 °C, and t3-LDPE_T 30 °C; and (II) all remaining samples. The increase in several encoding genes associated with plastic degradation in cluster I, like cutinase (K08095) and esterase/lipase (K01066), suggests a potential bacterial active response in soils containing plastic residues that responded positively to degradation [54, 56]. These enzymes are known to cleave the ester bonds and degrade polyurethane substrate, and therefore can act also on non-biodegradable plastics [55]. Cutinase can hydrolyze a broad variety of synthetic polymer esters, both soluble and insoluble, that are structurally related to cutin [56]. The increase of cellulose 1,4-beta-cellobiosidase (K01225, cluster I) that hydrolyze cellulose releasing cellobiose, was probably induced by the presence of biodegradable mulch residues in soil [57]. Cluster II comprises all samples at the latter sampling time, controls, and samples incubated at 45 °C. In particular, the presence of MB and TMB from later sampling times, where plastic degradation occurred, suggests that there may be significant differences in some metabolic activities compared to the same samples after 3 months of incubation. This confirms that changes in the activities of bacterial populations over time reflect the dynamics of plastic degradation in soil. Over time, polymers degrade enzymatically, and microorganisms assimilate and utilize the degradation products, potentially causing shifts in the predicted coding genes [20].

Finally, catalase performs an important role in soil ecosystem, and it can be used as a biological activity index to evaluate the quality of a particular soil [58, 59]. As previously indicated, variations of catalase-specific activities are also indicative of phylogenetic changes in community structure [60]. The tilling of biodegradable mulches modifies the soil ecosystem, promoting an environment in which aerobic bacteria thrive, which may have positive implications for soil health and quality.

### Core Microbiota Associated with Mulch Plastic Residues

The microbial core investigation aimed to provide hints of potential microorganisms involved in plastic breakdown for future investigations. The analysis was performed on all plastic-type soil systems at temperatures where breakdown occurred (RT and 30 °C). There is still a lack of literature, on information on microbial species known for their ability to degrade plastics. Thus, core members found in this work could be evaluated based on their enzymatic kit to assess their potential action on natural and non-natural polymers. Among the identified bacterial species, *Hydrogenispora ethanolica* LX-B isolated from the mesophilic (35 °C) anaerobic fermentation process of sludge can ferment substrates with different carbon sources, including starch, glucose, maltose, and fructose [61], suggesting its potential involvement in the degradation of starch-based plastics. The species *Thermoflavimicrobium daqui* FBKL4.01, a thermophilic bacterium isolated from the Daqu used to produce Chinese liquor Moutai, can ferments pure wheat [62], confirming its involvement in the degradation of starch and cellulose. The analysis of fungal core revealed 12 different species. Among them, *Solicoccozyma aeria* (*Cryptococcus aerius*) is known for its ability to produce amylases at 30 °C, to digest raw starch [63]. These three microbial species were found in soils amended with MB and TMB and could therefore be of interest for the biodegradation of starch-based plastics.

## Conclusions

Sustainable agricultural systems necessitate minimizing plastic waste accumulation in soils by transitioning to effective biodegradable alternatives. This study examines the impact of integrating both biodegradable and polyethylene mulch types into soil on the microbial community over a 6-month trial period, providing a direct comparison among mulch sheets applied under identical conditions and addressing a significant gap in the literature. The research observed a substantial decrease in the weight of Mater-Bi biodegradable plastics after 6 months of incubation, related to shifts in microbial community dynamics and bacterial functions under mesophilic conditions. Monitoring microbial responses offers valuable insights for optimizing agricultural management practices, thereby promoting sustainable and resilient agricultural systems. Moreover, the absence of plastic degradation observed when samples were incubated at 45 °C raises important considerations regarding potential applications under varying climate scenarios. Finally, through analysis of the core microbiota in plastic-influenced soil ecosystems, the study identifies microbial groups potentially pivotal in plastic biodegradation process.

### Supplementary Information

Below is the link to the electronic supplementary material.

Supplementary file1 (DOC 566 KB)

Supplementary file2 (XLSX 15.8 KB)

Supplementary file3 (XLSX 57.1 KB)

## Data Availability

The datasets presented in this study can be found in online repositories. The names of the repository and accession numbers can be found at PRJNA1127654.

## References

[1] Janssens V (2022) Plastics – the facts. In: PlasticsEurope AISBL. https:plasticseurope.org/de/wp-content/uploads/sites/3/2022/10/PE-PLASTICS-THE-FACTS_20221017.pdf

[2] Briassoulis D (2023) Agricultural plastics as a potential threat to food security, health, and environment through soil pollution by microplastics: problem definition. Sci Total Environ 892:164533. 10.1016/j.scitotenv.2023.164533 37285997 10.1016/j.scitotenv.2023.164533

[3] Büks F, Kaupenjohann M (2020) Global concentrations of microplastic in soils, a review. Soil Discuss 2020:1–26. 10.5194/soil-6-649-2020 10.5194/soil-6-649-2020

[4] Nizzetto L, Futter M, Langaas S (2016) Are agricultural soils dumps for microplastics of urban origin? Environ Sci Tech: 10777–10779. 10.1021/acs.est.6b04140 10.1021/acs.est.6b04140 27682621

[5] Koitabashi M, Noguchi MT, Sameshima-Yamashita Y et al (2012) Degradation of biodegradable plastic mulch films in soil environment by phylloplane fungi isolated from gramineous plants. AMB Express 2:1–10. 10.1186/2191-0855-2-40 22856640 10.1186/2191-0855-2-40 PMC3444367

[6] Li C, Moore-Kucera J, Miles C et al (2014) Degradation of potentially biodegradable plastic mulch films at three diverse U.S. locations. Agroecol Sustain Food Syst 38:861–889. 10.1080/21683565.2014.884515 10.1080/21683565.2014.884515

[7] Muroi F, Tachibana Y, Kobayashi Y et al (2016) Influences of poly(butylene adipate-co-terephthalate) on soil microbiota and plant growth. Polym Degrad Stab 129:338–346. 10.1016/j.polymdegradstab.2016.05.018 10.1016/j.polymdegradstab.2016.05.018

[8] Tian L, Jinjin C, Ji R et al (2022) Microplastics in agricultural soils: sources, effects, and their fate. Curr Opin Environ Sci Heal 25:100311. 10.1016/j.coesh.2021.100311

[9] Di Mola I, Cozzolino E, Ottaiano L et al (2023) Biodegradable mulching film vs. traditional polyethylene: effects on yield and quality of San Marzano tomato fruits. Plants 12:81030 10.3390/plants12183203 PMC10536419 37765367

[10] Bhattacharya S, Das S, Saha T (2018) Application of plasticulture in horticulture: a review. Pharma Innov J 7:584–585

[11] Di Mola I, Ventorino V, Cozzolino E et al (2021) Biodegradable mulching vs traditional polyethylene film for sustainable solarization: chemical properties and microbial community response to soil management. Appl Soil Ecol 163:103921. 10.1016/j.apsoil.2021.103921 10.1016/j.apsoil.2021.103921

[12] Ren X (2003) Biodegradable plastics: a solution or a challenge? J Clean Prod 11:27–40. 10.1016/S0959-6526(02)00020-3 10.1016/S0959-6526(02)00020-3

[13] Kyrikou I, Briassoulis D (2007) Biodegradation of agricultural plastic films: a critical review. J Polym Environ 15:125–150. 10.1007/s10924-007-0053-8 10.1007/s10924-007-0053-8

[14] AnonPlasticEurope (2021) Statistics: plasticulture in Europe. In: Plast. Facts 2021. APE Eur. website. https://apeeurope.eu/statistics

[15] Tsi H-Y, Tsen W-C, Shu Y-C et al (2009) Compatibility and characteristics of poly(butylene succinate) and propylene-co-ethylene copolymer blend. Polym Test 28:875–885. 10.1016/j.polymertesting.2009.08.004 10.1016/j.polymertesting.2009.08.004

[16] Castronuovo D, Candido V, Margiotta S et al (2005) Potential of a corn starch-based biodegradable plastic film for soil solarization. Acta Horticulturae. International Society for Horticultural Science (ISHS), Leuven, Belgium, pp 201–206

[17] Bandopadhyay S, Martin-Closas L, Pelacho AM, DeBruyn JM (2018) Biodegradable plastic mulch films: impacts on soil microbial communities and ecosystem functions. Front Microbiol 9:819. 10.3389/fmicb.2018.00819 29755440 10.3389/fmicb.2018.00819 PMC5932902

[18] Yamamoto-Tamura K, Hiradate S, Watanabe T et al (2015) Contribution of soil esterase to biodegradation of aliphatic polyester agricultural mulch film in cultivated soils. AMB Express 5:10. 10.1186/s13568-014-0088-x 25852987 10.1186/s13568-014-0088-x PMC4384995

[19] Wang D, Xi Y, Shi X et al (2023) Effects of residual plastic film on crop yield and soil fertility in a dryland farming system. J Integr Agric 22:3783–3791. 10.1016/j.jia.2023.04.026 10.1016/j.jia.2023.04.026

[20] Sander M (2019) Biodegradation of polymeric mulch films in agricultural soils: concepts, knowledge gaps, and future research directions. Environ Sci Technol 53:2304–2315. 10.1021/acs.est.8b05208 30698422 10.1021/acs.est.8b05208

[21] Pischedda A, Tosin M, Degli-Innocenti F (2019) Biodegradation of plastics in soil: the effect of temperature. Polym Degrad Stab 170:109017. 10.1016/j.polymdegradstab.2019.109017 10.1016/j.polymdegradstab.2019.109017

[22] DIN EN (2018) 17033: 2018–03 (E) Plastics—biodegradable mulch films for use in agriculture and horticulture—requirements and test methods. ISO Geneva, Switz

[23] Sünnemann M, Beugnon R, Breitkreuz C et al (2023) Climate change and cropland management compromise soil integrity and multifunctionality. Commun Earth Environ 4:394. 10.1038/s43247-023-01047-2 10.1038/s43247-023-01047-2

[24] Al Hosni AS, Pittman JK, Robson GD (2019) Microbial degradation of four biodegradable polymers in soil and compost demonstrating polycaprolactone as an ideal compostable plastic. Waste Manag 97:105–114. 10.1016/j.wasman.2019.07.042 31447017 10.1016/j.wasman.2019.07.042

[25] Bastioli C (1998) Properties and applications of Mater-Bi starch-based materials. Polym Degrad Stab 59:263–272. 10.1016/S0141-3910(97)00156-0 10.1016/S0141-3910(97)00156-0

[26] Kasirajan S, Ngouajio M (2012) Polyethylene and biodegradable mulches for agricultural applications: a review. Agron Sustain Dev 32:501–529. 10.1007/s13593-011-0068-3 10.1007/s13593-011-0068-3

[27] Ruggero F, Gori R, Lubello C (2019) Methodologies to assess biodegradation of bioplastics during aerobic composting and anaerobic digestion: a review. Waste Manag Res 37:959–975. 10.1177/0734242X19854127 31218932 10.1177/0734242X19854127

[28] Klindworth A, Pruesse E, Schweer T et al (2013) Evaluation of general 16S ribosomal RNA gene PCR primers for classical and next-generation sequencing-based diversity studies. Nucleic Acids Res 41:e1–e1. 10.1093/nar/gks808 22933715 10.1093/nar/gks808 PMC3592464

[29] Bokulich NA, Mills DA (2013) Improved selection of internal transcribed spacer-specific primers enables quantitative, ultra-high-throughput profiling of fungal communities. Appl Environ Microbiol 79:2519–2526. 10.1128/AEM.03870-12 23377949 10.1128/AEM.03870-12 PMC3623200

[30] Bolyen E, Rideout JR, Dillon MR et al (2019) Reproducible, interactive, scalable and extensible microbiome data science using QIIME 2. Nat Biotechnol 37:852–857. 10.1038/s41587-019-0209-9 31341288 10.1038/s41587-019-0209-9 PMC7015180

[31] Callahan BJ, McMurdie PJ, Rosen MJ et al (2016) DADA2: high-resolution sample inference from Illumina amplicon data. Nat Methods 13:581–583. 10.1038/nmeth.3869 27214047 10.1038/nmeth.3869 PMC4927377

[32] Kronthaler F, Zöllner S (2021) Testing hypotheses with RStudio BT - data analysis with RStudio: an easygoing introduction. In: Kronthaler F, Zöllner S (eds). Springer, Berlin, Heidelberg, pp 65–85. 10.1007/978-3-662-62518-7_6

[33] McMurdie PJ, Holmes S (2013) phyloseq: an R package for reproducible interactive analysis and graphics of microbiome census data. PLoS ONE 8:e61217. 10.1371/journal.pone.0061217 10.1371/journal.pone.0061217 PMC3632530 23630581

[34] Oksanen J, Blanchet FG, Friendly M et al (2019) vegan: community ecology package. R Packag version 2:1–2

[35] Bodenhausen N, Horton MW, Bergelson J (2013) Bacterial communities associated with the leaves and the roots of Arabidopsis thaliana. PLoS ONE 8:e56329. 10.1371/journal.pone.0056329 23457551 10.1371/journal.pone.0056329 PMC3574144

[36] Wang X, Hsu C, Dubeux JCB Jr et al (2019) Effects of rhizoma peanut cultivars (Arachis glabrata Benth.) on the soil bacterial diversity and predicted function in nitrogen fixation. Ecol Evol 9:12676–12687. 10.1002/ece3.5735 31788206 10.1002/ece3.5735 PMC6875664

[37] Gugliucci W, Cirillo V, Maggio A et al (2023) Valorisation of hydrothermal liquefaction wastewater in agriculture: effects on tobacco plants and rhizosphere microbiota. Front Plant Sci 14:1777. 10.3389/fpls.2023.1180061 10.3389/fpls.2023.1180061 PMC10277691 37342148

[38] Kolde R, Kolde MR (2015) Package ‘pheatmap’. R package 1(7):790

[39] Sintim HY, Bary AI, Hayes DG et al (2020) In situ degradation of biodegradable plastic mulch films in compost and agricultural soils. Sci Total Environ 727:138668. 10.1016/j.scitotenv.2020.138668 32334227 10.1016/j.scitotenv.2020.138668

[40] Santana MM, Gonzalez JM (2015) High temperature microbial activity in upper soil layers. FEMS Microbiol Lett 362:fnv182. 10.1093/femsle/fnv182 26424766 10.1093/femsle/fnv182

[41] Haider TP, Völker C, Kramm J et al (2019) Plastics of the future? The impact of biodegradable polymers on the environment and on society. Angew Chemie Int Ed 58:50–62. 10.1002/anie.201805766 10.1002/anie.201805766 29972726

[42] Campanale C, Galafassi S, Di Pippo F et al (2024) A critical review of biodegradable plastic mulch films in agriculture: definitions, scientific background and potential impacts. TrAC Trends Anal Chem 170:117391. 10.1016/j.trac.2023.117391 10.1016/j.trac.2023.117391

[43] Huang Y, Zhao Y, Wang J et al (2019) LDPE microplastic films alter microbial community composition and enzymatic activities in soil. Environ Pollut 254:112983. 10.1016/j.envpol.2019.112983 31394342 10.1016/j.envpol.2019.112983

[44] Li Y, Lin M, Ni Z et al (2020) Ecological influences of the migration of micro resin particles from crushed waste printed circuit boards on the dumping soil. J Hazard Mater 386:121020. 10.1016/j.jhazmat.2019.121020 31874765 10.1016/j.jhazmat.2019.121020

[45] Luo G, Jin T, Zhang H et al (2022) Deciphering the diversity and functions of plastisphere bacterial communities in plastic-mulching croplands of subtropical China. J Hazard Mater 422:126865. 10.1016/j.jhazmat.2021.126865 34449345 10.1016/j.jhazmat.2021.126865

[46] Wang J, Liu X, Dai Y et al (2020) Effects of co-loading of polyethylene microplastics and ciprofloxacin on the antibiotic degradation efficiency and microbial community structure in soil. Sci Total Environ 741:140463. 10.1016/j.scitotenv.2020.140463 32886986 10.1016/j.scitotenv.2020.140463

[47] Wolińska A, Kuźniar A, Zielenkiewicz U et al (2017) Bacteroidetes as a sensitive biological indicator of agricultural soil usage revealed by a culture-independent approach. Appl Soil Ecol 119:128–137. 10.1016/j.apsoil.2017.06.009 10.1016/j.apsoil.2017.06.009

[48] Partanen P, Hultman J, Paulin L et al (2010) Bacterial diversity at different stages of the composting process. BMC Microbiol 10:1–11. 10.1186/1471-2180-10-94 20350306 10.1186/1471-2180-10-94 PMC2907838

[49] Bhatia A, Madan S, Sahoo J et al (2013) Diversity of bacterial isolates during full scale rotary drum composting. Waste Manag 33:1595–1601. 10.1016/j.wasman.2013.03.019 23663960 10.1016/j.wasman.2013.03.019

[50] Debroas D, Mone A, Ter Halle A (2017) Plastics in the North Atlantic garbage patch: a boat-microbe for hitchhikers and plastic degraders. Sci Total Environ 599–600:1222–1232. 10.1016/j.scitotenv.2017.05.059 28514840 10.1016/j.scitotenv.2017.05.059

[51] Qian H, Zhang M, Liu G et al (2018) Effects of soil residual plastic film on soil microbial community structure and fertility. Water Air Soil Pollut 229:1–11. 10.1007/s11270-018-3916-9 10.1007/s11270-018-3916-9

[52] Abbate C, Scavo A, Pesce GR et al (2023) Soil bioplastic mulches for agroecosystem sustainability: a comprehensive review. Agriculture 13:197. 10.3390/agriculture13010197 10.3390/agriculture13010197

[53] Patel H, Rawat S (2021) Chapter 5 - Thermophilic fungi: diversity, physiology, genetics, and applications. In: Singh J, Gehlot PBT-N and FD in MB and B (eds). Elsevier, pp 69–93. 10.1016/B978-0-12-821005-5.00005-3

[54] Sharma H, Neelam DK (2023) Understanding challenges associated with plastic and bacterial approach toward plastic degradation. J Basic Microbiol 63:292–307. 10.1002/jobm.202200428 36470670 10.1002/jobm.202200428

[55] Bhardwaj H, Gupta R, Tiwari A (2013) Communities of microbial enzymes associated with biodegradation of plastics. J Polym Environ 21:575–579. 10.1007/s10924-012-0456-z 10.1007/s10924-012-0456-z

[56] Sahu S, Kaur A, Khatri M et al (2023) A review on cutinases enzyme in degradation of microplastics. J Environ Manage 347:119193. 10.1016/j.jenvman.2023.119193 37797518 10.1016/j.jenvman.2023.119193

[57] Kale SK, Deshmukh AG, Dudhare MS, Patil VB (2015) Microbial degradation of plastic: a review. J Biochem Technol 6:952–961

[58] Trasar-Cepeda C, Camina F, Leiros MC, Gil-Sotres F (1999) An improved method to measure catalase activity in soils. Soil Biol Biochem 31:483–485. 10.1016/s0038-0717(98)00153-9 10.1016/s0038-0717(98)00153-9

[59] Wang L, Kaur M, Zhang P et al (2021) Effect of different agricultural farming practices on microbial biomass and enzyme activities of celery growing field soil. Int J Environ Res Public Health 18:12862. 10.3390/ijerph182312862 34886587 10.3390/ijerph182312862 PMC8657710

[60] Chabot M, Morales E, Cummings J et al (2020) Simple kinetics, assay, and trends for soil microbial catalases. Anal Biochem 610:113901. 10.1016/j.ab.2020.113901 32841648 10.1016/j.ab.2020.113901

[61] Liu Y, Qiao J-T, Yuan X-Z et al (2014) Hydrogenispora ethanolica gen. nov., sp. nov., an anaerobic carbohydrate-fermenting bacterium from anaerobic sludge. Int J Syst Evol Microbiol 64:1756–1762. 10.1099/ijs.0.060186-0 24554637 10.1099/ijs.0.060186-0

[62] Li D, Huang W, Qiu S-Y (2019) Thermoflavimicrobium daqui sp. nov., a thermophilic microbe isolated from Moutai-flavour Daqu. Int J Syst Evol Microbiol 69:2709–2716. 10.1099/ijsem.0.003528 31310191 10.1099/ijsem.0.003528

[63] Shafiee R, Nahvi I, Emtiazi G (2005) Bioconversion of raw starch to SCP by co-culture of Cryptococcus aerius and Saccharomyces cerevisiae. J Biol Sci 5:717–723 10.3923/jbs.2005.717.723

