# Supplementary material for: Changes in Soil Microbial Communities Induced by Biodegradable and Polyethylene Mulch Residues Under Three Different Temperatures
Source: Microb Ecol. 2024 Jul 31;87:101. doi: 10.1007/s00248-024-02420-0 (PMC11291583; doi:10.1007/s00248-024-02420-0)
Supplement: Supplementary file 1 [file 248_2024_2420_MOESM1_ESM.doc]

Supplementary Material

**CHANGES IN SOIL MICROBIAL COMMUNITIES INDUCED BY BIODEGRADABLE AND POLYETHYLENE MULCH RESIDUES UNDER THREE DIFFERENT TEMPERATURES**

**Ida Romano^1^, Valeria Ventorino^1,2^, Mariachiara Schettino^1^, Giuseppina Magaraci^1^, Olimpia Pepe^1,2*^**

^1^Department of Agricultural Sciences, University of Naples Federico II, Naples, Italy

^2^Task Force on Microbiome Studies, University of Naples Federico II, Naples, Italy

*Corresponding author: [olipepe@unina.it](mailto:olipepe@unina.it)

***Materials and Methods***

***Determination of Soil chemical properties***

1–2 mg of fallow soil samples samples (n = 3) underwent combustion analysis by a Thermo Scientific Flash EA 1112, equipped with a thermal conductivity detector (TCD), to determine total C, H, N and S element contents. Calibration of the analyser, check of element accuracy and recovery were performed using acetanilide (Sigma Aldrich, 99.5%) standard. Organic carbon (OC) and organic matter (OM = OC 1.724) were determined by wet digestion using the Walkley-Black procedure. The pH was measured by potentiometry in milliQ water or 1 M KCl solution at 1/2.5 substrate/water ratio by HI 1131B pH meter (Hanna Instruments, Woonsocket, Rhode Island, USA), while electrical conductivity (EC) was measured at 1/5 substrate/water ratio by Basic 30 conductivity meter (Crison Instruments, Alella, Spain). The total carbonates were determined by gas-volumetric determination of CO_2_ released when the soil is treated with hydrochloric acid (HCl). Standard laboratory equipment included a Dietrich-Fruehling calcimeter, a thermometer for ambient temperature measurement, and a barometer.

***Results***

**Supplementary Table 1.** Percentage of degraded mulch residues (MB, TMB, LDPE) after 6 months of incubation in soil (t6) at different temperatures (RT, T30°C, T45°C). Different letters indicate statistically significant differences (univariate ANOVA and Tuckey's HSD post hoc, P < 0.05).

| **Percentage (%) of degraded mulching residuals** | | | |
| --- | --- | --- | --- |
|  | **RT** | **30°C** | **45°C** |
| **MB** | 69.15±13.12^ab^ | 88.90±1.70^a^ | 5.31±3.80^c^ |
| **TMB** | 51.36±12.64^ab^ | 38.86±17.18^bc^ | 0.00±0.00^c^ |
| **LDPE** | 3.01±1.85^c^ | 0.07±0.07^c^ | 3.56±1.95^c^ |

*Values represent the means ± SE of five replicates*

**Supplementary Table 2.** Analysis of Variance of Shannon Index for bacteria (A), fungi (B), on the plastic type, temperature and sampling time.

| **A** | **Df** | **Sum Sq** | **Mean Sq** | **F value** | **Pr(>F)** | **Sign.** |
| --- | --- | --- | --- | --- | --- | --- |
| **Mulch Plastic Type** | 3 | 1142931 | 380977 | 4.5726 | 0.006458 | ** |
| **Temperature** | 2 | 634075 | 317038 | 3.8052 | 0.028689 | * |
| **Sampling Time** | 2 | 51244 | 25622 | 0.3075 | 0.736593 |  |
| **Residuals** | 52 | 4332505 | 83317 |  |  |  |
| **Significance:** 0 ‘***’ 0.001 ‘**’ 0.01 ‘*’ 0.05 ‘.’ 0.1 ‘ ’ 1 | | | | | | |

| **B** | **Df** | **Sum Sq** | **Mean Sq** | **F value** | **Pr(>F)** | **Sign.** |
| --- | --- | --- | --- | --- | --- | --- |
| **Mulch Plastic Type** | 3 | 3892.9 | 1297.63 | 2.6725 | 0.056260 | . |
| **Temperature** | 2 | 5665.2 | 2832.60 | 5.8338 | 0.005039 | ** |
| **Sampling Time** | 2 | 269.6 | 134.78 | 0.2776 | 0.758670 |  |
| **Residuals** | 55 | 26705.3 | 485.55 |  |  |  |
| **Significance:** 0 ‘***’ 0.001 ‘**’ 0.01 ‘*’ 0.05 ‘.’ 0.1 ‘ ’ 1 | | | | | | |

**Supplementary Table 3.** Output of PERMANOVA analysis for bacteria (A). and fungi (B) on the plastic type. temperature and sampling time based on Beta diversity assessed with Bray–Curtis distance.

| **A** | **Df** | **SumOfSqs** | **R2** | **F value** | **Pr(>F)** | **Significatività** |
| --- | --- | --- | --- | --- | --- | --- |
| **Mulch Plastic Type** | 3 | 18.020 | 0.07735 | 17.662 | 0.023 | * |
| **Temperature** | 2 | 20.249 | 0.08691 | 29.770 | 0.001 | *** |
| **Sampling Time** | 2 | 14.462 | 0.06207 | 21.262 | 0.020 | * |
| **Residuals** | 36 | 180.245 | 0.77367 |  |  |  |
| **Total** | 60 | 232.975 | 100.000 |  |  |  |
| **Significance:** 0 ‘***’ 0.001 ‘**’ 0.01 ‘*’ 0.05 ‘.’ 0.1 ‘ ’ 1 | | | | | | |

| **B** | **Df** | **SumOfSqs** | **R2** | **F value** | **Pr(>F)** | **Significatività** |
| --- | --- | --- | --- | --- | --- | --- |
| **Mulch Plastic Type** | 3 | 12.272 | 0.08803 | 39.507 | 0.001 | *** |
| **Temperature** | 2 | 61.435 | 0.44067 | 296.673 | 0.001 | *** |
| **Sampling Time** | 2 | 0.8758 | 0.06282 | 42.291 | 0.001 | *** |
| **Residuals** | 55 | 56.947 | 0.40848 |  |  |  |
| **Total** | 62 | 139.412 | 100.000 |  |  |  |
| **Significance:** 0 ‘***’ 0.001 ‘**’ 0.01 ‘*’ 0.05 ‘.’ 0.1 ‘ ’ 1 | | | | | | |

**Supplementary Table 4** Results of the analysis of variance (ANOVA) conducted to assess the variations of relative abundance of bacterial and fungal phyla on the plastic type. temperature and sampling time.

Excelfile “Supplementary_Table_4”.

**Supplementary Table 5** Results of the analysis of variance (ANOVA) conducted to assess the variations in predicted enzyme-encoding functional genes on the plastic type. temperature and sampling time. The dataset used for the analysis consisted of catalase, cutinase, cellobiosidase, hydrolase and lipase enzyme‐encoding genes predictions obtained through Tax4fun R package.

Excelfile “Supplementary_Table_5”.

**Supplementary Figure 1**. Alpha diversity of bacterial, communities in soil samples containing Mater-Bi (MB), biodegradable plastic (TMB) and non-biodegradable plastic (LDPE) mulch films at the beginning (t0), after three (t3) and six (t6) months of incubation at different temperatures (room temperature RT, 30°C, 45°C). Control soil samples without plastic (C) were analyzed in parallel. Asterisks denote statistical significant differences given by T-test (0 ‘***’ 0.001 ‘**’ 0.01 ‘*’ 0.05 ‘.’ 0.1 ‘ ’ 1).

**Supplementary Figure 2** Predicted abundances of catalase genes predicted through Tax4fun R package. The color code refers to gene abundance, with high predicted abundances (blue) and low predicted abundances (light yellow) in soil samples containing Mater-Bi (MB), biodegradable plastic (TMB) and non-biodegradable plastic (LDPE) mulch films at the beginning (t0), after three (t3) and six (t6) months of incubation at different temperatures (room temperature RT, 30°C, 45°C). Control soil samples without plastic (C) were analyzed in parallel.

**Supplementary Figure 3**. Venn diagram of the bacterial (A) and fungal (B) community calculated on the basis of the value of the relative abundance of common and uncommon taxa among the soil samples containing Mater-Bi (MB). biodegradable plastic (TMB) and non-biodegradable plastic (LDPE) mulch films incubated at room temperature and at 30°C. The core was calculated by considering the dominant taxa in 70% of the samples (detection >0.01%) for bacteria and 99% of the samples (detection >0.1%) for fungi.
